# Motor styles in action: Developing a computational framework for operationalization of motor distances

**DOI:** 10.3758/s13428-024-02530-0

**Published:** 2024-12-11

**Authors:** Jordi Manuello, Camilla Maronati, Matilde Rocca, Riccardo Guidotti, Tommaso Costa, Andrea Cavallo

**Affiliations:** 1 Move’N’Brains Lab, Department of Psychology, University of Turin, Via Verdi, 10, 10124 Turin, Italy; 2 FOCUS Lab, Department of Psychology, University of Turin, Turin, Italy; 3 Social Intelligence Lab, Department of Psychology and Berlin School of Mind and Brain, Humboldt University of Berlin, Berlin, Germany

**Keywords:** Procrustes transformation, Kinematics, Motor distance

## Abstract

Aside from some common movement regularities, significant inter-individual and inter-trial variation within the same individual exists in motor system output. However, there is still a lack of a robust and widely adopted solution for quantifying the degree of similarity between movements. We therefore developed an innovative approach based on the Procrustes transformation to compute 'motor distance' between pairs of kinematic data. As a proof of concept, we tested this on a dataset of reach-to-grasp movements performed by 16 participants while acting with the same confederate. Using the information of wrist velocity, acceleration, and jerk, the proposed technique was able to correctly estimate smaller distances between movements performed by the confederate compared with those of participants. Moreover, the reconstructed pattern of inter-subject distances was consistent when computed either on precision grip prehension or whole hand prehension, suggesting its suitability for the investigation of 'motor styles'. The definition of a solid approach to 'motor distance' computation, therefore, opens the way to new research lines in the field of movement kinematics.

## Introduction

A growing number of psychophysical studies, especially those leveraging the analysis of movement kinematics, show the existence of high inter-individual variability in motor system output (Ting et al., 2015; Todorov & Jordan, 1998). From a mechanistic point of view, at least part of this variability can be attributed to the high redundancy of the musculoskeletal system, which is estimated to have 244 degrees of freedom (Prilutsky & Zatsiorsky, 2002). Biometric differences among people (e.g., their height, weight, limb length) should also be taken into account (de C. Hamilton et al., 2004). Moreover, sources of variability could also be found at the level of movement planning, both in the form of different movement approaches used to perform a task (Cowin et al., 2022; Dhawale et al., 2017) and in the form of different neural strategies used to control the implementation of a motor task (Avrillon et al., 2021). The combination of these elements makes highly unlikely to exactly replicate an action under identical environmental conditions and circumstances and therefore observe any two different individuals adopting the same postural configuration to complete a given task (Vidal & Lacquaniti, 2021). However, motor variability is not only observed on the inter-individual level, but also on the intra-individual one. This is particularly evident when analyzing multiple trials performed by a same subject during an experimental session and is considered a fundamental characteristic of biological behavior (Faisal et al., 2008). While this can be seen as an undesired effect of noise which prevents the precise repetition of action planning or execution, variability is also a way to explore a space of possible solutions, supporting for example the processes of learning a new movement (Dhawale et al., 2017), relearning motor skills after brain damage (DePaul et al., 2015; Hornby et al., 2019; Park et al., 2021; Ziegler et al., 2010) and modifying or adjusting a motor response to fit with social or non-social environmental demands (i.e., [social]fitting) (Casartelli et al., 2023; Ciceri et al., 2023). Intriguingly, it has been suggested that the amount of inter-trial variability could be itself a characteristic feature of an individual (Wu et al., 2014). Although in consequence of intra-individual variability, each person displays a range of alternative solutions for the same movement rather than a unique version, this range was found to be relatively stable (Haar et al., 2017) (for a comprehensive theoretical model of motor variability, see Casartelli et al., 2023). Moreover, and more importantly, despite intra-individual variability, inter-individual variability remains marked enough to suggest the existence of what could be defined as the construct of 'motor style'. This aspect seems to reflect also on the perceptual side. In fact, individuals make more accurate perceptual predictions when they observe their own actions rather than those of others (Knoblich & Flach, 2001), and the same holds true for observed actions falling within the own domain of motor experience (Abernethy et al., 2008; Cross et al., 2006; Jackson et al., 2006).

The construct of 'motor style' still lacks a unanimous definition, however. In their recent and pivotal review, Vidal and Lacquaniti (2021) reported the weighty definition of style made by Ernst Gombrich as “any distinctive, and therefore recognizable, way in which an act is performed” (Preziosi, 1998). Notably, this definition links the execution of an action with its understanding, and gains support from many empirical data in the field of cognitive psychology and cognitive neuroscience that highlight the pivotal role of the motor system on an individual's capacity to predict the outcome of observed actions (e.g., Bonini et al., 2013; Koul et al., 2018; Soriano et al., 2018). Nevertheless, any practical use of the theoretical concept of 'motor style' for research cannot do without a translation into a computational framework. To facilitate quantitative analysis of 'motor styles', it is essential to first identify metrics that capture the range of motor solutions within an individual and then offer a quantifiable measure of 'motor distance' between individuals. Going into this direction, Słowiński and colleagues (2016) used the Earth mover’s distance (EMD) (Levina & Bickel, 2001) to compare the profiles of hand velocity of different subjects performing a motor task under different experimental conditions. Interestingly, they found that couples of subjects with a smaller EMD between them are facilitated in achieving coordination during interaction. However, due to the modeling through histograms of frequencies, this technique does not consider the temporal profile of the action. As a further drawback, this approach is based on the information of a single kinematic variable. Consequently, the use of a different parameter could potentially lead to different results. 'Individual motor style' is supposed to capture and be informed by a wider range of features, and it would be desirable to consider combinations of them when computing 'motor distance'. Hilt and colleagues (2020) have also attempted to compute an 'individual motor signature distance' (IMS), but like the EMD method, the IMS method only allows the use of a single kinematic variable at a time to describe movements. Other approaches often used to compare movements features are correlational (De Marco et al., 2020) or based on the use of Dynamic Time Warping (DTW) (Sakoe & Chiba, 1978; Ullah & Finch, 2013), which also allows to derive a measure of distance between the compared time series. Bearing in mind the complexity of defining 'motor distance' (or similarity) in a universal way, implying that the operationalization of this concept is likely to be adapted depending on the context investigated and on the nature of the available data, here we have developed a computational approach based on the Procrustes transformation technique, which allows us to combine a theoretically unlimited number of kinematic features. The Procrustes transformation, a method for comparing shapes and landmarks, is per se a technique already established in the field of motor control. It has been applied for shape characterization (Veeraraghavan et al., 2004) or as a tool to investigate differences in movements inherently dissimilar due to their nature or environment (i.e., distinct classes of movements or the same class but performed in different contexts) (Adams & Cerney, 2007; Passos et al., 2017). It has also been employed to compute distances between trials, with the purpose of identifying computational costs or invariants in movement repetitions (Haggard et al., 1995; Haggard & Richardson, 1996; Kadmon Harpaz et al., 2014). Furthermore, it has been used for preprocessing alignment (Alshabani et al., 2007) or for defining a 'normal mean shape' as a norm for subsequent analyses (Anwary et al., 2019; Wong et al., 2019). All these attempts went, in various ways, in the direction of computing a similarity index between movements.

The aim of the present work is to extend the current use of the Procrustes transformation in motor control research by optimizing it as a computational approach to identify motor (dis)similarities and provide a reliable metric of 'motor distance' between individuals, with a specific interest for upper-limb reach-to-grasp movements. To do so, we first describe how we have applied the Procrustes transformation in the development of the approach. Then, we show how our method was used to compute the intra-subject and inter-subject motor distances between different experimental sessions of a reach-to-grasp task. Since, as stated before, intra-subject variability is expected to be smaller than inter-subject one, we hypothesized to be able to reconstruct smaller motor distance between sessions performed by a same subject compared with sessions performed by different subjects.

## Materials and methods

### Participants

The present work is based on the dataset previously acquired and described by Rocca and colleagues (2023). Briefly, this consists of 16 right-handed participants (nine female; age range = 25–40 years; mean age = 29.06 ± 4.34 years), with normal or corrected-to-normal vision, and no history of neurological disorders. Complete data were also collected for the only confederate (male, 35 years) who interacted with each of the subjects during the experimental procedure. This particular aspect made it the ideal input for the focus of the current research. All participants had originally provided written informed consent in accordance with the principles of the revised Helsinki Declaration (World Medical Association, 2013) and were naïve with respect to the purpose of the experiment.

### Experimental procedure and dataset

**Fig. 1:**
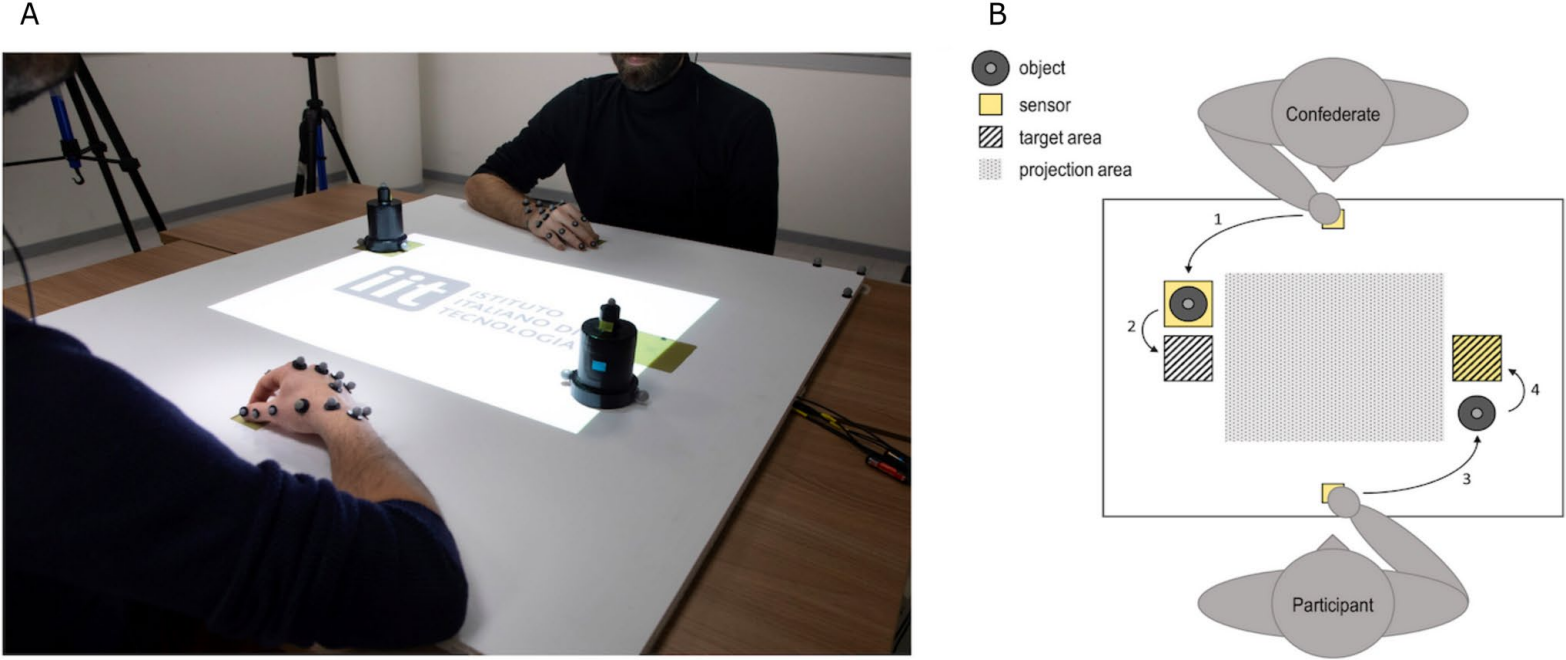
Experimental setup. Panel **A** shows a photo of the experimental setup, in which the participant and the confederate keep their hands in their respective starting positions. In front of the two agents are placed the two objects used during the experiment. The objects were designed to be grasped with either a precision grip or a whole-hand prehension. Panel **B** shows a schematic (not in scale) representation of the experimental setup. The numbers refer to the order in which the actions were performed during each motor sequence (i.e., trial). Press and release sensible sensors were placed in strategic positions to control for correct performance during each trial Adapted from Rocca et al. (2023)

The dataset used for the main analyses of this paper is composed of kinematic data of the non-interactive condition described in Rocca et al. (2023) (see Sect. 1 of the supplementary information for details concerning the additional conditions and how these were used for control analyses). In that condition, the participant and the confederate sat at opposite sides of a table facing each other. The experiment was explained to them as a simple sequential individualistic task. The general rule of the task was to reach and grasp an object consisting of two superimposed cylinders with different diameters (upper part: height = 3 cm, Ø = 2.5 cm; lower part: height = 10 cm, Ø = 5.5 cm) that was in front of them, and to place it on the target area as quickly and accurately as possible. Participants could start moving only after the confederate had lifted his own object (Fig. 1). Depending on instructions, the objects had to be grasped with either a precision grip (PG) or a whole-hand prehension (WHP). For the present main analyses, only the 320 trials (20 trials × 16 participants) in which both the confederate and the subjects performed PG were considered; further 320 trials of WHP were used as control condition (see supplementary information).

Twenty retro-reflective hemispheric markers of 6 mm in diameter were placed on both agents’ right hands. Movement kinematics were recorded using eight near-infrared cameras for motion-capture (frame rate: 100 Hz; Vicon Nexus v.2.5). Each collected trial was individually inspected for correct marker labelling; a low-pass Butterworth filter with an 8 Hz cutoff was then applied. Trials in which the quality of marker reconstruction was poor, and trials in which either the confederate or the participant performed wrong or inaccurate movements were excluded from subsequent analyses. For each of the 254 PG trials finally retained, the following variables were computed for both agents:

- Wrist velocity (WV), defined as the module of wrist velocity (mm/s)
- Wrist acceleration (WA), defined as the rate of change of wrist velocity (mm/s^2^)
- Wrist jerk (WJ), defined as the rate of change of the module of wrist acceleration (mm/s^3^)

In order to allow inter-trial comparison irrespective of their duration, the time window from onset to offset of the reach-to-grasp phase of the movement was divided into 10 time points. A moving average with no overlap was then computed over deciles, so that, for example, the value of WV at the first time point represents the mean WV measured during the first 10% of the movement time, and so on (for a similar approach see Koul et al., 2018; Montobbio et al., 2022). Although a wide variety of kinematics variables can be computed, these were selected because of their transversality across motor tasks, either transitive or intransitive. In fact, while grip aperture or finger plane are only informative for reaching or manipulation movements, WV, WA and WJ are suitable to describe any kind of trajectory, therefore allowing the extension of this approach to different experimental scenarios.

### The Procrustes transformation

To quantify the motor distance between individuals we propose an approach based on the Procrustes transformation. In general terms, this technique aims to find the optimal transformation to be applied to the so-called "comparison matrix" to minimize its distance to the so-called "target matrix" (Bookstein, 1992). This approach is commonly used to compare shapes and their landmarks, but it can be extended to whichever dataset allows the representation of its elements as points distributed in an *n*-dimensional space. By applying a combination of translation, scaling, and reflection the Procrustes algorithm generates the “transformed matrix”, being the closest match with the target matrix while preserving the original shape of the comparison matrix. The remaining distance between the transformed and the target matrices can then be computed as the sum of squared differences between the corresponding points in the two distributions. This is called the Procrustes distance. In the present work, the Procrustes method was used as implemented in the MATLAB function “procrustes”, allowing translation, scaling, and reflection. Notably, the possibility to compute a distance index while also generating a transformed version of the input is a peculiarity of Procrustes technique that distinguishes it from other methods commonly used in kinematics research.

### 'Motor distance' computation

The procedure to compute the motor distance between subjects consisted of two steps described below: an initial inter-trial alignment and the actual inter-subject distance computation.

#### Inter-trial alignment

**Fig. 2:**
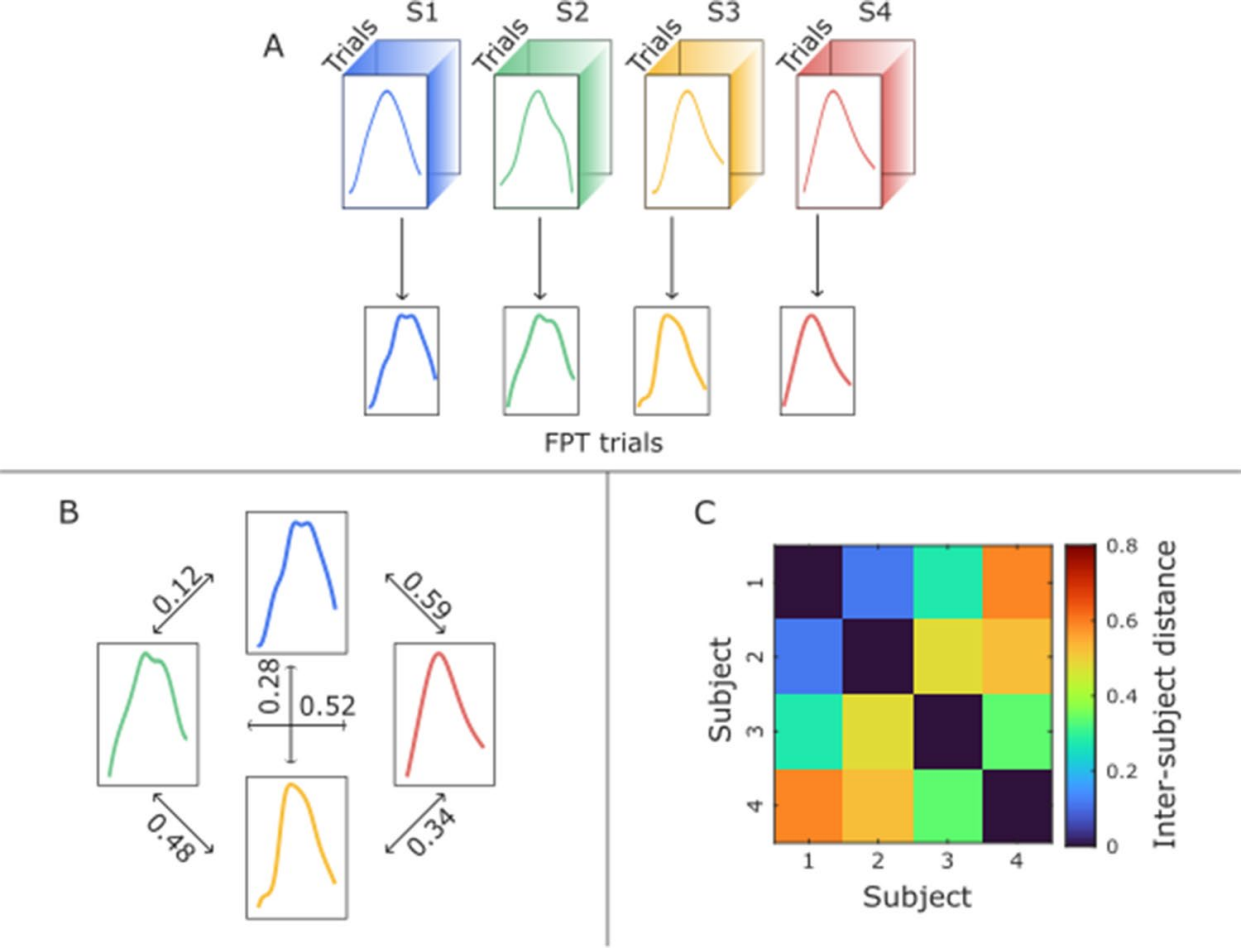
Schematic representation of the steps implemented to compute the motor distance. Panel **A** depicts the inter-trial alignment procedure used to create each subject’s final Procrustes-transformed (FPT) trial. In the panels at the bottom there are two examples of the second step of the procedure: Panel **B** shows the resulting distances between six pairs of individuals, and Panel **C** is a schematic representation of the distance matrix obtained at the end of the procedure from the distances shown in Panel **B**

As mentioned before, each participant performed multiple PG trials (for the analyses on WHP trials, see the supplementary information). However, the final computation of inter-subject motor distance required each subject to be represented by a single reach-to-grasp instance. Therefore, in order to retain the information of inter-trial variability, an average trial was obtained using Procrustes transformation. To do so, each trial was represented through a 10 × 3 matrix, storing the value of each of the three measured variables at each of the 10 time points. In other words, this is equivalent to placing the 10 time points in a three-dimensional space defined by the axes WV, WA, WJ. To refer to the traditional Procrustes logic, each trial can be seen as a shape described by 10 landmarks (i.e., the time points). For each participant, a given executed trial was taken as the target matrix. The Procrustes transformation was then applied to the remaining trials (i.e., the comparison matrices) to minimize the distance of each of them to the selected trial. Therefore, one transformed matrix was obtained for each target–comparison couple, providing the WV–WA–WJ coordinates of the transformed time points. These were stored in a 10 × 3 × *t* matrix, where *t* is the number of trials retained after quality assessment for each participant. Finally, a unique final Procrustes-transformed (FPT) trial (i.e., a 10 × 3 matrix) was obtained averaging across the *t* dimension. Standard deviation was also computed at each of the 10 time points, capturing the variability across the *t* trials. The same procedure was also applied to the data about the confederate, treating it as a different subject for each interaction with a different participant. Therefore, the inter-trial alignment produced 32 FPT trials, 16 referred to the participants, and 16 referred to the confederate interacting with them (Fig. 2A). For the main analyses described, the first available trial for each participant was used as target matrix. However, to exclude possible biases induced by this arbitrary selection, the analyses were repeated choosing different trials for this role (see Sects. 2 and 3.1 of supplementary information).

#### Inter-subject distance computation

The 32 FPT trials were then used to quantify the motor distance between subjects, considering both the 16 participants and the 16 instances of the confederate. Therefore, one FPT trial was fixed as the target matrix, and the remaining 31 were iteratively used as comparison matrix. For each iteration, the Procrustes distance was computed between the transformed matrix and the target matrix, as the sum of squared differences between the corresponding points in the two distributions. Therefore, a 32 × 32 symmetric distance matrix was obtained, covering all the possible confederate–confederate, participant–participant, and confederate–participant couplings (Fig. 2B, C). Of note, distances returned by the Procrustes function in MATLAB are by default in the range 0–1 and were therefore kept so. We hypothesized that if our approach could correctly estimate the motor distance, smaller values should be observed overall for confederate–confederate couples than for confederate–participant couples. To statistically test this, an empirical *p*-value was computed considering the chance (over 1000 permutations) to reduce the average confederate–confederate motor distance after replacing the distance value of a random confederate–confederate couple with a random confederate–participant couple. We ensured that for each iteration of the permutation process, the first confederate from the confederate–confederate matrix was replaced by the corresponding confederate from the confederate–participant matrix. Additionally, in order to gain more insight about the internal functioning of the Procrustes method, and to improve naturalistic interpretability of results being produced by it, a comparison of kinematic variables for subjects deemed more similar or dissimilar was performed as follows. First, one column of the previously obtained 32 × 32 distance matrix was randomly selected and sorted by increasing distance. This encodes the motor distance between a given reference and the remaining participants or instances of the confederate. Then, WV, WA, and WJ were plotted for the reference, its closest subject, the 33rd percentile, the 66th percentile, and the most dissimilar subject. Note that these kinematic variables refer to the FPT trial obtained at the end of the inter-trial alignment.

### Multidimensional scaling of inter-subject motor distances

To confirm whether the proposed approach actually captured motor distance, the 32 × 32 distance matrix was used as input for a two-dimensional multidimensional scaling (MDS) (separately for PG and WHP). The aim of this technique is to represent a set of elements originally distributed in an *n*-dimensional space (with *n* often being unknown) into a new system with reduced dimensionality, preserving the spatial relationships between the elements. Here, the 2-dimensions MDS was initialized with a principal coordinate axes approach (Torgerson, 1958) as implemented in Orange v2.7 (Demsar et al., 2013). Kruskal stress (Kruskal, 1964) was used to evaluate the goodness of the optimal solution. If our approach correctly estimates motor distance, we expected to observe a good segregation between the 16 participants and the 16 instances of the confederate. Moreover, the latter should appear less scattered than the others.

### Robustness analysis: ICC absolute agreement

The proposed approach to the computation of motor distance operates, in its final stage, on a single FPT trial resulting from several repetitions of reach-to-grasp movements. It is therefore relevant to quantify how the number of repetitions considered for the analysis influences the obtained FPT. To investigate this, the following procedure was implemented. First, several subsamples of movements were generated removing an increasing percentage of them from the dataset (from 10% to 80%, at steps of 10%). For each step of subsampling, a random selection of movements was repeated 1000 times. Then, motor distance was computed for each of the 8000 random selections obtained (1000 iterations × 8 subsampling percentages) as described in Sect. 2.4. This produced eight matrices of size 32 × 32 × 1000, which were dimensionally reduced by averaging across the third dimension, so as to obtain a mean distance matrix for each percentage of removed trials. To quantify the similarity between each of the eight reduced matrices and the original one representing the complete dataset we estimated, using intraclass correlation coefficients (McGraw & Wong, 1996), the absolute agreement, i.e., the extent to which each of the reduced matrices and the original matrix yield similar absolute values of motor distance.

### Comparison with IMS method

In order to compare the performance of the proposed method with an approach already proposed in the literature, data were processed following the individual motor signature (IMS) method described in Hilt et al. (2020). Briefly, this technique compares the similarity between subjects by computing the root mean squared error (RMSE) between one kinematic variable at a time of those used to describe the movement. Then, the various distance matrices are normalized and averaged together to obtain a single representative one. It should be noted that the Procrustes transformation allows instead to jointly model all the variables of interest, therefore taking better into account the possible interactions among them. In order to compare the performance of the two methods, the ground truth was defined considering that the various instances of the confederate are all expressions of the same subject. Consequently, the most accurate method should be the one that estimates the minimal average distance among those instances. This was quantified as the delta between the average distance for the confederate–confederate couples obtained through the two procedures. To test the statistical significance of this delta, the following permutation approach was implemented. First, a cell from the Procrustes distance matrix was substituted by the corresponding cell in the matrix containing the results of the Hilt method. Then, the mean distance for the modified Procrustes matrix was computed. This procedure was repeated for each of the 120 cells (i.e., the upper triangular part of the symmetric matrix excluding its diagonal), each time restarting from the original matrix so to replace only one value at a time. Finally, an empirical *p*-value was obtained counting the fraction of cases in which moving a value obtained through the Hilt method into the Procrustes distance matrix reduced the mean motor distance originally computed.

To better characterize the behavior of the two methods, the same permutation approach was applied also to the confederate–participant couples and to the participant–participant couples (note that in the latter case, given the asymmetric nature of the matrix, 256 iterations were completed).

### Cross-condition agreement

Finally, in order to test the sensitivity of motor distance to different motor tasks, the results obtained for PG condition and WHP condition were compared. Specifically, the two 32 × 32 distance matrices were used as input for the Mantel test (Mantel, 1967). This technique allows to correctly compute the correlation between two matrices by means of a permutation test (5000 in this case), each time randomly shuffling the order of rows and columns. The empirical *p*-value for statistical significance is then derived on this basis.

## Results

### 'Motor distance'

**Fig. 3:**
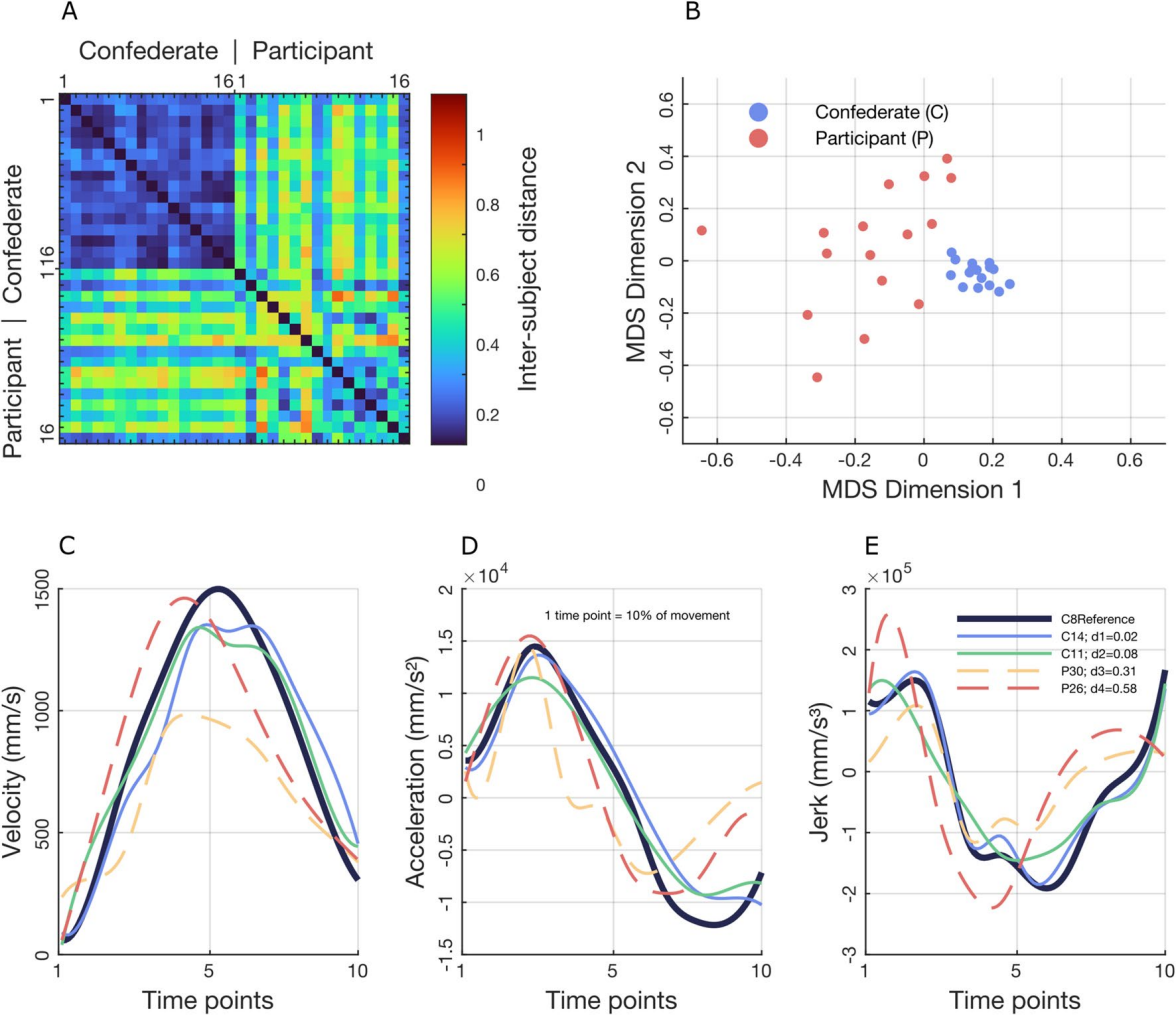
Results of the inter-subject motor distance computation for PG movements. Panel **A** shows the 32 × 32 symmetric distance matrix covering all the possible confederate–confederate, participant–participant, and confederate–participant pairs. A smaller motor distance is given for confederate–confederate pairs than for confederate–participant pairs. Panel **B** shows the two-dimensional MDS; good separation is shown between confederate occurrences and participants. Panels from **C** to **E** show velocity, acceleration, and jerk profiles for the reference subject (in dark blue), nearest subject (in light blue), 33rd percentile (in green), 66th percentile (in yellow), and the most dissimilar subject (in red). Each time point on the *X*-axis corresponds to a 10% of the reach-to-grasp movement. The plotted data have been interpolated (100 values instead of 10) only for display purposes

To verify whether motor distance between subjects can be objectively quantified, a combination of Procrustes transformations was applied to a set of upper limb movements (see Sect. 4 of the supplementary information for the plot of the obtained FPT trials). Coherently with our hypothesis, smaller distances were observed for confederate–confederate couples than for confederate–participant couples (Fig. 3A). In fact, the distances for the confederate–confederate condition ranged between 0.01 and 0.21, with an average of 0.09. For the confederate–participant condition, the range was 0.16–0.63, with an average of 0.39. The empirical *p*-value obtained over 1000 iterations was 0.023 (see Sect. 3.2 of supplementary information for the results of WHP condition). Overall, visual inspection confirmed that subjects deemed to be highly similar also showed comparable velocity, acceleration, and jerk across the 10 time points (Fig. 3C, E) (see Sect. 3.3 of supplementary information for the results of WHP condition).

### Multidimensional scaling

The 2-dimensions MDS based on the computed inter-subject motor distances confirmed that the confederate instances and the actual participants were well segregated (Fig. 3B). Moreover, the 16 instances of the confederate remained spatially concentrated, while the 16 participants were more spread. The optimal solution was obtained after 65 steps with a value of Kruskal stress = 0.004 (see Sect. 3.4 of supplementary information for the results of WHP condition).

### Robustness analysis

**Fig. 4:**
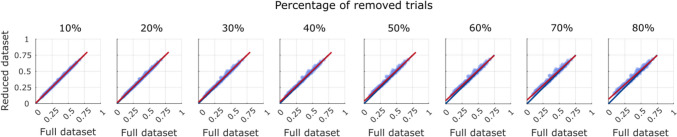
Scatterplot of the inter-individual motor distances for PG movements computed with the full dataset (*X* axes) against the inter-individual motor distances computed with the reduced datasets (*Y* axes). Each plot depicts a reduced dataset with a different percentage of removed trials, from 10% to 80%. Each dot represents the inter-individual distance between a confederate–participant pair. The blue line represents the theoretical line of equality between distances computed with the full and the reduced dataset. The red line is the least-squares line passing through the observed data. ICCs ranged between 0.958 (80% of removed trials) and 0.999 (10% of removed trials)

The ICC results showed an absolute agreement of 0.999 when the 10% of the sample was removed, indicating near-perfect agreement between the absolute values of inter-individual motor distance computed using all the trials, and those computed removing 10% of the trials. Even if slightly reduced, absolute agreement remained close to 1 also when 80% of the trials were removed to compute the inter-individual motor distance (ICC = 0.958; Fig. 4) (see Sect. 3.5 for the replication on WHP trials).

### Comparison with IMS method

The approach designed by Hilt and colleagues (cit.) returned an average distance of 0.204 for the confederate–confederate couples, while the Procrustes method returned an average distance of 0.088 (note that in both cases, values range between 0 and 1). Notably, none of the permutations produced a confederate–confederate distance smaller than that obtained using the Procrustes method. On the contrary, the difference between the average motor distances obtained through the two methods was not found to be statistically significant for confederate–participant couples (empirical *p*-value = 0.051) and participant–participant couples (empirical *p*-value = 0.275).

### Cross-condition agreement

The Mantel test computed between the PG and the WHP distance matrices returned a correlation value of 0.78 (empirical-*p* after 5000 permutations < 1 × 10^−6^), meaning that, in most cases, the motor distance between a given couple is consistently computed despite the different kind of grip used by the subjects.

## Discussion

**Fig. 5:**
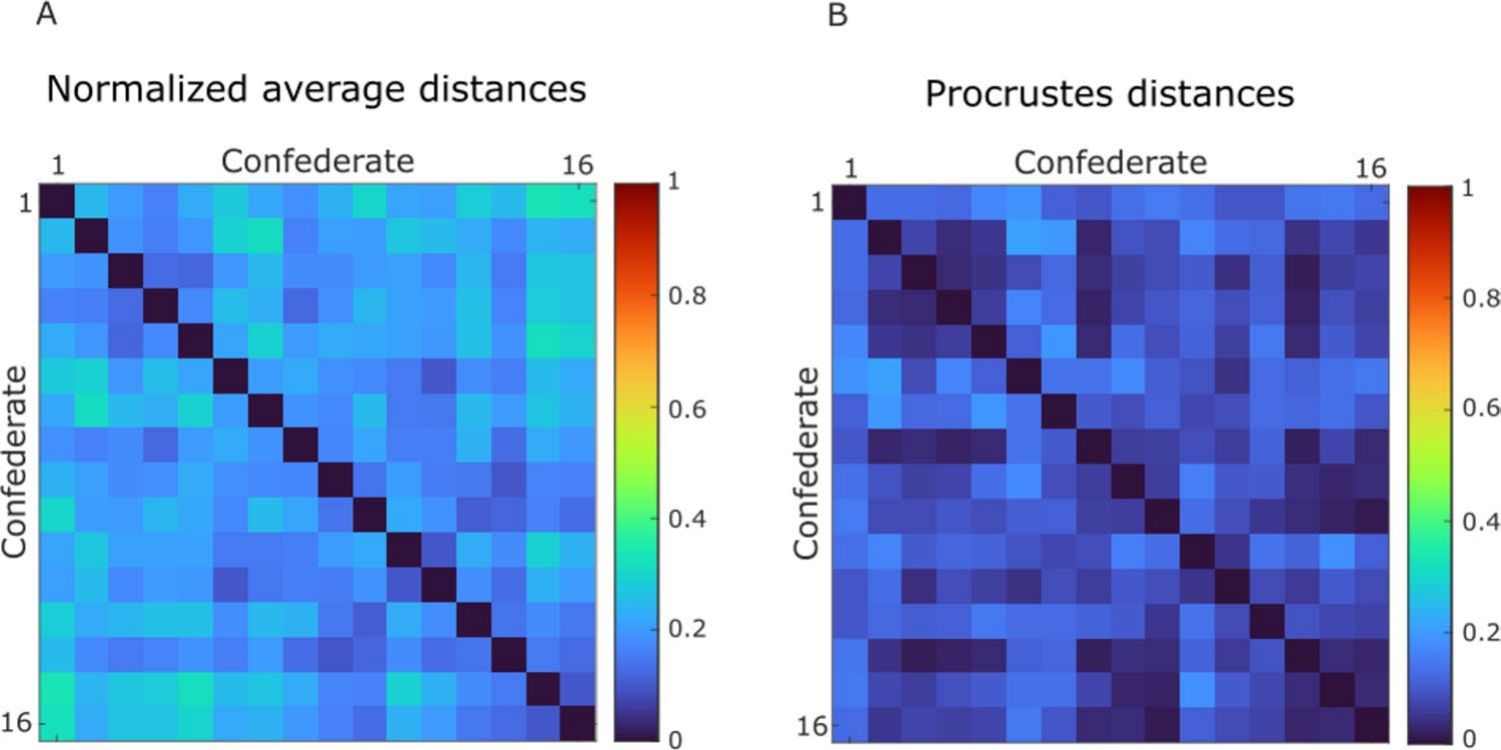
Distance matrix between the confederate instances computed through the Hilt et al., 2020 method (panel **A**) and Procrustes transformation (panel **B**)

While it is empirically recognized that individuals move in ways that are either similar or dissimilar between each other, movement and kinematics research has not yet established a standardized method to quantify this. Ideally, given that the intricacy of human movement cannot be fully captured by a single variable, such a method should encompass multiple kinematic features simultaneously and should consider the temporal evolution of each feature during the action. With this in mind, we developed a computational approach based on the Procrustes transformation. A relevant characteristic of this technique is the possibility to both transform data and compute their distance to a reference at the same time. However, the actual meaning of a distance in the domain of motor behavior is far from being straightforward. If subject A is seated at 0.5 m from subject C, and subject B is seated at 1.0 m from subject C, the fact that A is closer to C becomes obvious and no particular explanation of this spatial relationship is needed. But what if we say that A has a motor distance of 0.5 from C while B has 1 instead? How can we be sure that this relationship is actually capturing something meaningful? As mentioned in the introduction, we set as ground truth that (neat to a certain degree of within-subject variability) a subject tends to move more similarly to himself than to other people. Consequently, we hypothesized that if our approach can correctly estimate motor distance, smaller (and the smallest possible) values should be observed when comparing movements made by the same person. We therefore resorted to a previously collected motion capture dataset (Rocca et al., 2023) that allowed us to directly test our hypothesis in virtue of a large number of movements performed by a same person (i.e., the one who acted as confederate for the whole experimental campaign), together with data for the 16 participants. Results confirmed our hypothesis, showing that the motor distances computed between couples of instances of the confederate (blue top left quarter of the distance matrix in Fig. 3A) are significantly smaller than those computed between the confederate and the participants (top right quarter of the distance matrix in Fig. 3A). Moreover, the comparison with the IMS method (Hilt et al., 2020) showed that the proposed procedure is able to estimate smaller distances for the confederate–confederate couples (Fig. 5). The fact that no significant difference emerged for the confederate–participant and participant–participant couples suggests that the Procrustes method does not estimate smaller values per se, but only when small motor distances actually exist. These results indicate that the method is precise in this context, but further testing across different tasks and datasets is needed to confirm its broader applicability.

It should be noted that the confederate and each participant performed the task sitting in front of each other, although the task required no active interaction between them. This means that the motor distances we obtained in the confederate–participant couples were reduced by the motor interference effects that usually arise when two agents do not share a common goal (Clarke et al., 2019; Sacheli et al., 2018, 2019). Therefore, the observed delta of 0.39 between the average motor distance in the confederate–participant conditions could be an underestimation of the real magnitude. Having said this, not all the small distances were limited to the confederate–confederate condition (e.g., blue stripes appear also in the top right quarter of the distance matrix in Fig. 3A). Therefore, while the proposed method was able to capture the whole picture by isolating the various instances of the confederate, it was also sensitive to a range of confederate–participant distances. The visual inspection of the trajectories (Fig. 3C, E) also confirmed that, although imagining a movement plotted in a velocity × acceleration × jerk space could be not particularly straightforward, the evaluation made by the Procrustes transformation is easily intelligible. In fact, the plotted variables clearly show differences in amplitudes, peaks, and shapes. At the same time, those plots also verify that the FPT trials obtained at the end of the inter-trial alignment (see Sect. 2.3.1) preserve a human-like appearance, although those movements had never been stricto sensu performed. Notably, the robustness analysis showed that the method is stable enough to allow reliable application even with just four repetitions of the movement for each subject.

Finally, the very high coherence observed between the distance matrices of the PG and the WHP conditions opens the way to a quantitative cross-modal definition of 'motor styles'. A crucial aspect of the operationalization of 'motor distance' is in fact to make it consistent across movements, otherwise subjects A and B could be very close when grasping a bottle but not at all when pointing to something, making it difficult to conceive the 'motor style' as a person’s trait. While PG and WHP are indeed quite similar movements, we showed how the proposed approach allows the comparison of different movements, and ad hoc tasks and datasets could be designed in the future towards this goal.

### Limitations and future directions

The dataset used was fruitfully instrumental in testing the proposed approach. At the same time, it also potentially introduced some bias due to the conjoint presence of the confederate and the participant during the task, although without any kind of cooperation or goal-oriented interaction. However, as mentioned before, there is no reason to expect this to have either systematically reduced the variability among confederate instances or increased the delta between confederate–confederate and confederate–participant comparisons. At the same time, the control analyses performed on the further conditions available in the same dataset allowed the replication of the effect even in case of real interaction. As a further limitation of the dataset, it should be noted that while it offered a comprehensive representation of transitive movements through power and precision grip, the application of the proposed Procrustes transformation-based approach to different kinds of movements (e.g., intransitive and cyclic ones) still remains to be explored in future research. This may require a subtle and accurate tuning of the method to fit the specific requirements of the analyzed data. The investigation of new scenarios will provide further information concerning the generalizability of quantified 'motor distance' toward identifying a stable metric of 'individual motor style' that might be independent of the specific class of movements performed by an individual. As a third point, we did not explore in depth the participant–participant couples, as they were not instrumental to test our current hypotheses. However, future studies could use the proposed approach to investigate which features are related to 'motor distance' (e.g., age, height, sex, anthropometric characteristics, social proximity). Moreover, it will be now possible to study the potential modulatory effect of 'motor distance' on movement observation, as in the event of a better recognition of the size of a hidden object by watching the reaching movement towards it when the observer and the executer are closer (in motor terms). This line of reasoning could also be extended to the investigation of pathologies characterized by an alteration of kinematics performance or kinematics interpretation (or both) as in the case of autism spectrum disorder (ASD). Finally, in the present work the Procrustes transformation was applied to obtain a unique FPT trial deriving from several repetition of movements performed by the subject. However, the same technique could be instrumental to quantify the distance between each single movement, allowing therefore to explore the intra-individual variability, that has been recently suggested to be a relevant component of the 'individual motor style' (Casartelli et al., 2023; Haar et al., 2017).

## Conclusion

Determining the degree of similarity between the way in which different people execute a same movement is of relevance for several research fields. Here, we proposed a method to compute 'motor distance' based on the Procrustes transformation algorithm. The obtained results proved that this method can both identify subjects’ peculiarities and capture various degrees of similarities, opening therefore the way to several research lines.

## Data Availability

The database used for the analyses presented is freely available from https://osf.io/htdn3/?view_only=f70023d1a997415da58faf5025419b1d

## Code availability

The code used for the analyses presented can be downloaded from https://github.com/Jordi-Manuello/motor_distance

## Declarations

### Open practice statement

Data and code are made freely available for other colleagues.
